# Percutaneous nephroscopy versus flexible ureteroscopy in the treatment of calyceal diverticulum calculi: a meta-analysis

**DOI:** 10.1186/s12894-024-01655-w

**Published:** 2025-01-02

**Authors:** Conglei Hu, Rui Ma, Yongxiang Shao, Zilong Liang, Meng Cheng, Haofeng Pang, Liping Yao, Fei Liu

**Affiliations:** 1 Department of Urology, Xijing Hospital, Air Force Medical University, Xi’an, 710032 China; 2 Department of Geriatrics, Xijing Hospital, Air Force Medical University, Xi’an, 710032 China; 3 Graduate Department, Xi’an Medical University, Xi’an, 710021 China; 4 National Clinical Research Center for Digestive Diseases and Xijing Hospital of Digestive Diseases, Air Force Medical University, Xi’an, 710032 China

**Keywords:** Calyceal diverticulum calculi, Percutaneous nephrolithotomy, Flexible ureteroscopic lithotripsy, Retrograde intrarenal surgery, Meta-analysis

## Abstract

**Background:**

There is still controversy about the best minimally invasive surgical method for the treatment of calyceal diverticulum calculi. We conducted meta-analysis to evaluate the effectiveness and safety of PCNL and FURL in the treatment of calyceal diverticulum calculi.

**Methods:**

We searched Pubmed, Cochrane Library, Web of Science, Embase, Clinical trial platform, CNKI, VIP until April 2024. We utilized the Newcastle–Ottawa Scale (NOS, 0 to 9 stars) to assess the quality of the included literature.

**Results:**

Totally 15 high-quality studies with 755 patients were included in the meta-analysis. Meta-analysis showed that FURL group was better than PCNL group in blood loss [SMD = 1.713, 95%CI:(0.858, 2.568), *Z* = 3.928, *P* = 0.000] and hospital stay [SMD = 2.611, 95%CI: (1.726, 3.496), *Z* = 5.784, *P* = 0.000], there was no significant difference in operating time [SMD = 0.079, 95%CI:(-0.43, 0.589), *Z* = 0.306, *P* = 0.760], complication rate [OR = 1.793,95%CI: (0.952,2.602), *Z* = 1.586, *P* = 0.113], stone-free rate [OR = 1.339, 95%CI: (0.576, 3.112), *Z* = 0.678, *P* = 0.497] and symptom-free rate [OR = 3.826,95%CI: (0.561,10.238), *Z* = 0.966, *P* = 0.334] as well.

**Conclusion:**

Whether FURL is indeed superior to PCNL in safety, whether FURL's efficacy is really close to PCNL, and whether FURL can surpass PCNL as the first choice for the treatment of renal diverticulum stones in the future need to be further verified by multi-center, large-sample and high-quality studies.

**Supplementary Information:**

The online version contains supplementary material available at 10.1186/s12894-024-01655-w.

## Introduction

Calyceal diverticulum (CD) is a relatively rare congenital renal anomaly. It occurs when the ureteral bud fails to retract from the third or fourth branch of the Wolffian duct [1, 2]. This condition leads to a non-secretory cavity within the kidney parenchyma, which is lined with urothelium. A calyceal diverticulum lacks draining renal papillae. It features a narrow neck that connects to the renal collecting system. This narrow neck allows urine to passively drain into the diverticulum [3, 4]. Calyceal diverticulum was first described by Rayer in 1841 and diagnosed by intravenous urography with an incidence of 0.21–0.45% in adults. Stone formation within the diverticulum has been reported in 9.5–50% of cases. These stones can lead to symptoms such as pain and hematuria. Patients can also experience recurrent urinary tract infections and damage to the surrounding parenchyma [5, 6]. As the disease progresses, patients with calyceal diverticulum stones may develop serious complications such as infection of perirenal tissue [7].

Most calyceal diverticula are asymptomatic and may not require surgical intervention. However, treatment is recommended when calyceal diverticulum calculi are associated with pain, recurrent infections, hematuria, or decreased renal function. Extracorporeal shock wave lithotripsy (ESWL) shows the advantages of low cost and minimally invasive management, but its stone clearance rate (SCR) is only 4–20% [8]. Laparoscopic and robotic surgery are indicated for refractory stones located in the anterior group of renal calyces with heavy load and unsuccessful treatment by other minimally invasive surgical methods. Although the above two surgical methods can obtain high SCR, they are the most invasive in minimally invasive treatment, and may lead to severe renal parenchyma damage in some cases, and put forward high requirements for the surgical skills of the operator [9–11]. With the advancement of endourological techniques, flexible ureteroscopy (FURL) and percutaneous nephrolithotomy (PCNL) are becoming the first choice for the treatment of calyceal diverticulum calculi [3, 12]. However, due to the different characteristics and indications of percutaneous nephrolithotomy and flexible ureteroscopy, there is still controversy about the best minimally invasive surgical method for the treatment of calyceal diverticulum calculi [13]. Therefore, this study collected and sorted out the published comparative research literatures on PCNL and FURL in the treatment of calyceal diverticulum calculi up to April 2024. The present study conducted systematic analysis by meta analysis method, and objectively evaluated the effectiveness and safety of PCNL and FURL in the treatment of calyceal diverticulum calculi, hoping to provide evidence-based reference for clinical treatment of calyceal diverticulum calculi.

## Method

### Literature selecting strategies

We searched Pubmed, Cochrane Library, Web of Science, Embase, Clinical trial platform (clinicaltrial.gov), CNKI, VIP, Wanfang Data according to predefined search criteria. We searched in all fields using the search formula “Caliceal diverticular calculi” OR “Calyceal diverticulum stone” OR “Caliceal diverticulum calculi” OR “Renal calyceal diverticulum” OR “Renal caliceal diverticulum” OR “Kidney calyceal diverticulum” OR “Calyceal pouch” OR “Calyceal diverticulum” OR “Diverticular calculi” OR “Diverticulum stones” OR “Diverticular stones”. The Chinese database was searched using the same search formula. Searching relevant articles from the establishment of each database to April 2024, regardless of language. In addition, relevant references searches were continued to improve the recall of included studies.

### Inclusion and exclusion criteria

The inclusion criteria for this study are as follows: (a) the type of study included in the original research article must be a controlled clinical trial; (b) subjects: imaging (CT, IVP, B-ultrasound) diagnosed as calyceal diverticulum calculi; (c) intervention measures: FURL and PCNL were used to treat calyceal diverticulum calculi respectively, the operation was traditional surgical procedure without using improved technology, holmium laser was used for lithotripsy; (d) efficacy indicators: stone-free rate, symptom-free rate, hospital stay, blood loss, operating time and complication rate.

Exclusion criteria for this study were as follows: (a) comparative study of non-PCNL versus FURL for the treatment of calyceal diverticulum stones; (b) ambiguous literature, unable to extract corresponding data and results; (c) basic research, animal experiments, conference abstracts, letters, editorials, reviews, case reports; (d) stones in other parts of the urinary system in addition to calyceal diverticulum calculi requiring surgical treatment; (e) preoperative diverticulum ablation. For studies with overlapping data reporting results, only the study with the largest sample size was selected.

### Data extraction

To avoid bias during data extraction, all eligible studies were extracted by two independent investigators, including publication year, first author, country, patient baseline information, stone size, stone-free rate, symptom-free rate, length of hospital stay, blood loss, operating time and complication rate. In case of disagreement between the two investigators, the third investigator was consulted or relevant experts were consulted for adjudication.

### Quality evaluation

All eligible studies were non-randomized studies. The Newcastle–Ottawa Scale (NOS, ranging from 0 to 9 stars) was employed to assess the quality of the included literature based on study population selection, inter-group comparability, and exposure or outcome evaluation. Studies scoring 6 or higher were categorized as high quality, while those scoring 5 or lower were deemed to be of poor quality (https://www.ohri.ca/programs/clinical_epidemiology/oxford.asp) [14].

### Statistical methods

Meta analysis was performed after data extraction, using SPSSAU platform (www.spssau.com). Continuous variables were statistically analyzed using standard mean difference (SMD), and binomial variables were statistically analyzed using effect odds ratio (OR) after pooling. 95% confidence interval (CI) was selected for each effect. I^2^ was used for heterogeneity analysis. When I^2^ < 50%, it indicated that the heterogeneity of the included studies was low or not obvious. Fixed effects model could be used for data pooling. The results were expressed as forest plots; When the heterogeneity test I^2^ > 50%, it indicates that the heterogeneity of the included studies is obvious, and the random effect model can be used for statistical analysis after data pooling. Robustness was analyzed by sensitivity testing, using a one-by-one elimination approach, omitting one study per round for sensitivity analysis. The potential publication bias of this study was estimated by Begg’s test and Egger’s test. The report of this study was conducted and reported according to the Preferred Reporting Item of the Guidelines for Systematic Review and Meta-Analysis (PRISMA) [15]. *P* < 0.05 was statistically significant.

## Results

### Literature selection and characteristics

**Fig. 1:**
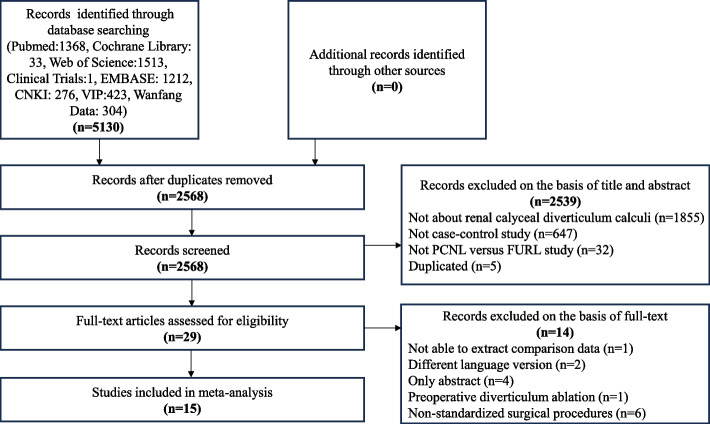
Flow chart of articles retrieval

According to the search strategy, 5130 articles were screened (Pubmed 1368, Cochrane Library 33, Web of Science 1513, Embase 1212, Clinical trial platform 1, CNKI 276, VIP 423, Wanfang data 304). A total of 2562 duplicate articles were removed, leaving 2568. After screening titles and abstracts, a total of 2539 articles were excluded, and the remaining 29 articles were re-screened. After full-text screening of 29 articles, 14 articles were excluded because full-text was not available, there were no outcome indicators, or there were not enough extractable data. Ultimately, our study included 15 articles [3, 4, 16–28] involving a total of 755 patients, 386 patients in the PCNL group and 369 patients in the FURL group. A flowchart of the selection process is shown in Fig. 1.

The studies included were published between 2015 and 2023 and their main characteristics and clinical results are summarized in Table 1. The methodological assessment of NOS ranged from 6 to 8, indicating that all studies in our meta-analysis were of high quality (Table 2).

**Table 1:** Main characteristics of the eligible studies

Author	Year	Country	Technique	Sample size	Age (years)	Gender	Type	Stone size (cm)	Location	Outcome indicator
Bas [3]	2015	Türkiye	PCNL	29	45.10 ± 10.53	14:15	NA	211 ± 97(mm^2^)	4:7:18	③④⑤⑥
			FURL	25	36.28 ± 10.43	12:13	NA	154 ± 77(mm^2^)	12:8:5	
Fan [16]	2015	China	PCNL	29	39.93 ± 9.80	15:14	NA	2.14 ± 0.50	5:10:14	①③④⑤⑥
			FURL	26	38.76 ± 8.60	12:14	NA	1.60 ± 0.40	14:8:4	
Luo [17]	2015	China	PCNL	22	54.31 ± 11.95	NA	NA	1.74 ± 0.36	7:9:6	①②③④
			FURL	19	57.05 ± 10.86	NA	NA	1.60 ± 0.25	4:8:7	
Wang [18]	2017	China	PCNL	44	55.17 ± 9.68	NA	NA	1.58 ± 0.28	NA	①②③
			FURL	38	55.21 ± 9.67	NA	NA	1.57 ± 0.29	NA	
Zhang [19]	2017	China	PCNL	17	45.00 ± 12.34	7:10	NA	2.22 ± 0.46	9:4:4	①③④⑤⑥
			FURL	12	44.08 ± 15.68	3:9	NA	1.96 ± 0.26	8:3:1	
Luo [20]	2018	China	PCNL	26	NA	NA	NA	NA	NA	④⑥
			FURL	26	NA	NA	NA	NA	NA	
Cai [21]	2019	China	PCNL	25	48.48 ± 11.10	17:8	NA	1.62 ± 0.22	NA	①②③④⑤
			FURL	25	49.12 ± 11.54	15:10	NA	1.69 ± 0.15	NA	
Ding [22]	2019	China	PCNL	20	38 ± 10	NA	NA	1.7 ± 0.5	NA	①②③④⑤
			FURL	18	35 ± 14	NA	NA	1.5 ± 0.4	NA	
Liu [23]	2020	China	PCNL	34	50.50 ± 22.09	18:16	NA	NA	NA	①②③④
			FURL	34	51.50 ± 22.07	19:15	NA	NA	NA	
Pang [24]	2021	China	PCNL	22	50.73 ± 10.58	8:14	NA	2.02 ± 0.36	NA	①③④⑤
			FURL	24	50.42 ± 11.38	11:13	NA	1.86 ± 0.26	NA	
Wang [25]	2021	China	PCNL	9	44.44 ± 13.53	3:6	NA	1.38 ± 0.62	9:0:0	①③④⑤⑥
			FURL	9	47.89 ± 12.82	4:5	NA	1.35 ± 0.49	6:3:0	
Zeng [4]	2022	China	PCNL	12	44.5 ± 20.3	5:7	NA	1.9 ± 1.4	6:2:4	①②③④⑤
			FURL	14	39.6 ± 11.4	5:9	NA	1.7 ± 0.9	8:2:4	
Zhu [26]	2022	China	PCNL	42	46.8 ± 2.3	20:22	NA	NA	27:10:5	①②③④⑤
			FURL	44	47.2 ± 2.5	21:23	NA	NA	26:11:7	
Zong [27]	2022	China	PCNL	30	45.71 ± 12.52	20:10	NA	NA	NA	①②③⑤
			FURL	30	45.31 ± 12.22	13:17	NA	NA	NA	
Liu [28]	2023	China	PCNL	25	34.56 ± 8.99	15:10	NA	NA	NA	④⑤
			FURL	25	33.67 ± 8.96	14:11	NA	NA	NA	

Gender, Male:Female

Type, diverticulum type, Type I:Type II

Location, stone location, upper pole: mid kidney: lower pole; *NA* not applicable

Outcome indicators: ①operating time; ②blood loss; ③hospital stay; ④complication rate; ⑤stone-free rate; ⑥symptom-free rate

**Table 2:** Newcastle–Ottawa score of eligible studies

**Study**		**Selection**			**Comparability**		**Exposure**		**Score**
	Definition	Representativeness	Selection^a^	Definition^a^	Minimum:2	Ascertainment	Same method	Non-Response rate	
Bas 2015 [3]	1	1	1	1	1	1	1	0	7
Fan 2015 [16]	1	1	1	1	1	1	1	0	7
Luo 2015 [17]	1	1	1	1	1	1	1	0	7
Wang 2017 [18]	1	1	1	1	2	1	1	0	8
Zhang 2017 [19]	1	0	1	1	1	1	1	0	6
Luo 2018 [20]	1	1	1	1	1	1	1	0	7
Cai 2019 [21]	1	1	1	1	2	1	1	0	8
Ding 2019 [22]	1	1	1	1	1	1	1	0	7
Liu 2020 [23]	1	1	1	1	2	1	1	0	8
Pang 2021 [24]	1	1	1	1	1	1	1	0	7
Wang 2021 [25]	1	0	1	1	1	1	1	0	6
Zeng 2022 [4]	1	0	1	1	1	1	1	0	6
Zhu 2022 [26]	1	0	1	1	1	1	1	0	6
Zong 2022 [27]	1	1	1	1	2	1	1	0	8
Liu 2023 [28]	1	1	1	1	1	1	1	0	7

^a^Controls’ selection and definition

### Meta-analysis results

#### Operating time

**Fig. 2:**
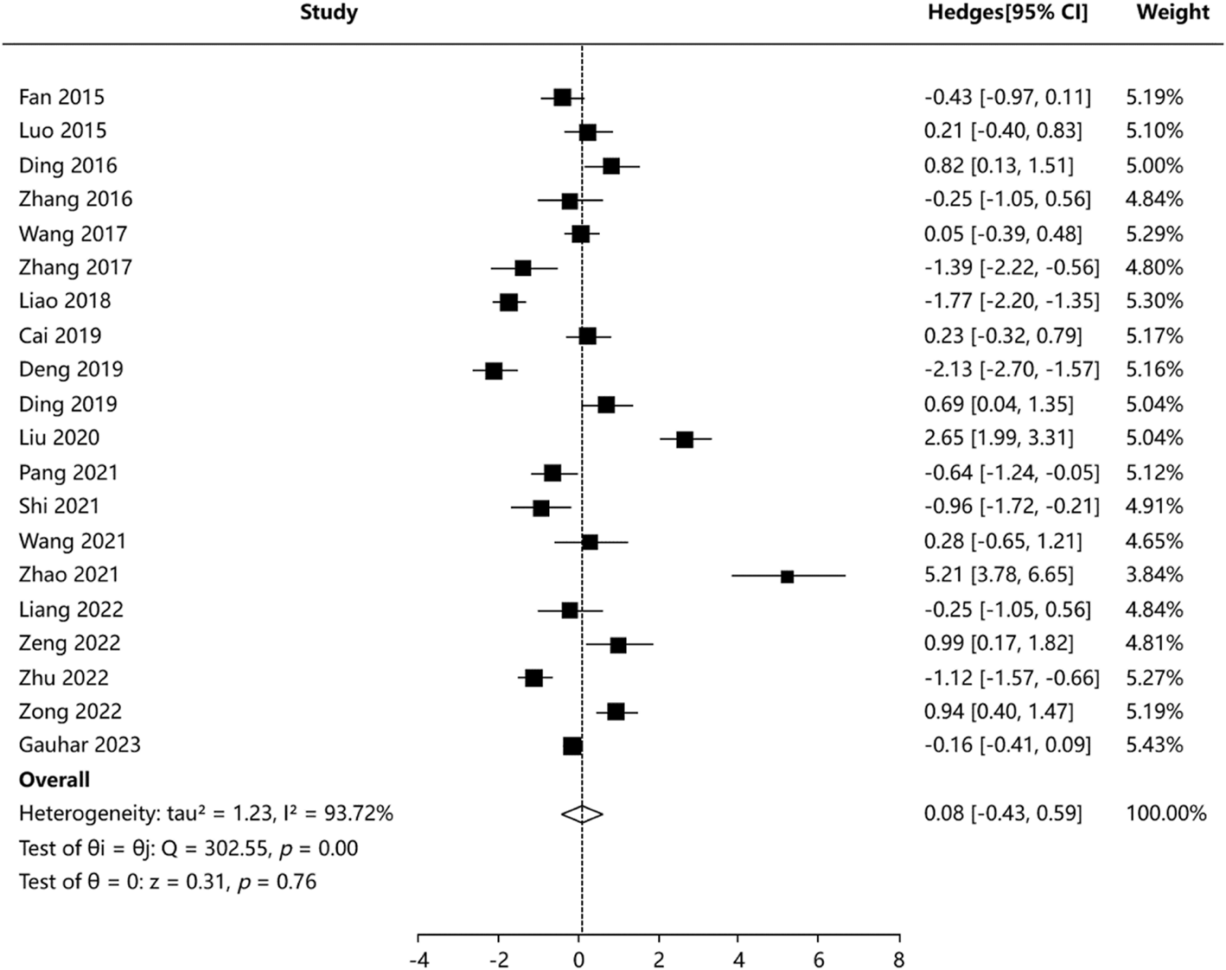
Forest plot showing pooled SMD with 95% CI of operation time

Twelve articles [4, 16–19, 21–27] reported operating time, and the results of heterogeneity test of articles were (*P* = 0.000, I^2^ = 93.72%), indicating that the heterogeneity of included articles was significant. Random-effects model was applied for meta-analysis. The results showed that there was no statistically significant difference in operating time between PCNL group and FURL group: SMD = 0.079, 95%CI:(−0.43, 0.589), *Z* = 0.306, *P* = 0.760 (Fig. 2). Publication bias test including Egger’s test *P* = 0.180, Begg’s test *P* = 0.205, indicating no publication bias. The combined effect value adjusted by Trim’s method is 95%CI:(−0.922,0.207). The above conclusion still holds true. Sensitivity analysis results indicated that the results for pooled effects were generally robust. Funnel plots of publication bias test and forest plot of sensitivity analysis were showed in Figure S1.

#### Blood loss

**Fig. 3:**
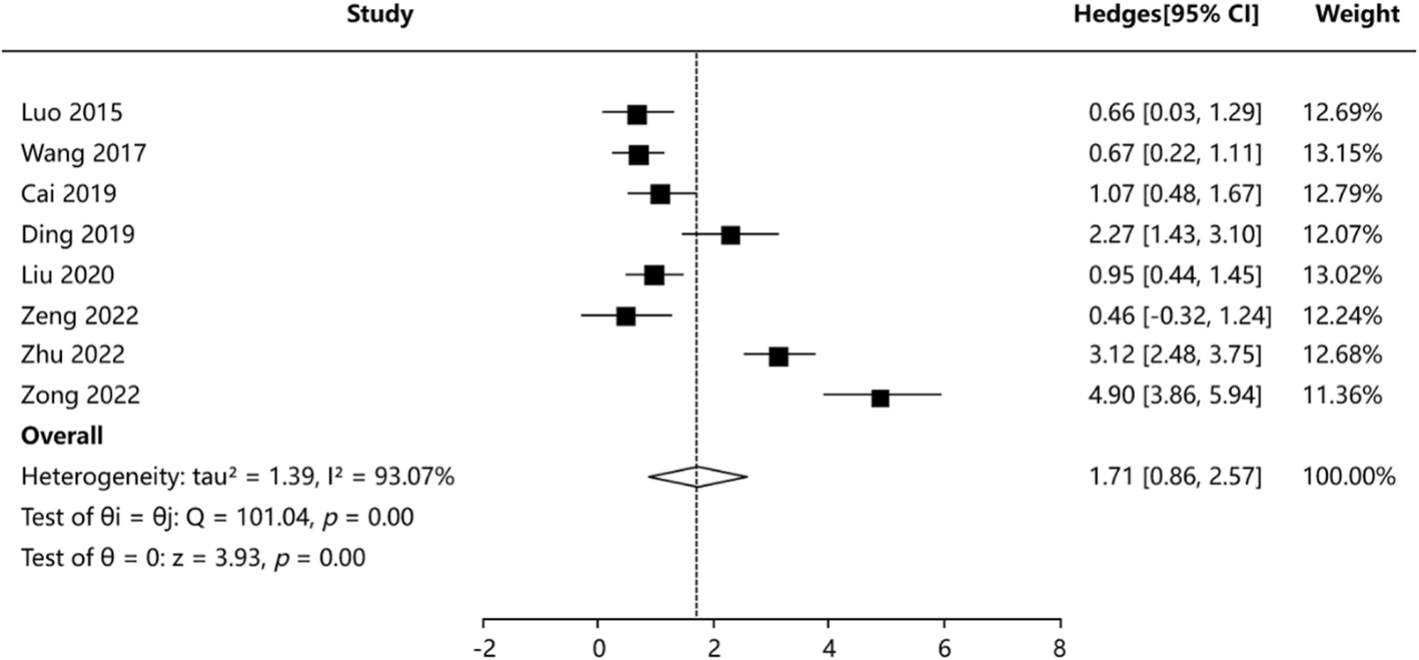
Forest plot showing pooled SMD with 95% CI of blood loss

Eight articles [4, 17, 18, 21–23, 26, 27] reported intraoperative blood loss, and the results of heterogeneity test were (*P* = 0.000, I^2^ = 93.07%), indicating that the heterogeneity of the included articles was significant. Random-effects model was applied for meta-analysis. The results showed that there was a statistically significant difference in intraoperative blood loss between PCNL group and FURL group: SMD = 1.713, 95%CI:(0.858, 2.568), *Z* = 3.928, *P* = 0.000 (Fig. 3). A statistically significant difference may indicate that intraoperative blood loss was shorter in the FURL group than in the PCNL group. Publication bias test including Egger’s test *P* = 0.084, Begg’s test *P* = 0.083, indicating no publication bias. We utilized Trim’s method to adjust combined effect value. The combined effect value adjusted by Trim’s method is 95%CI:(0.319,2.243). The above conclusion still holds true. Sensitivity analysis results indicated that the results for pooled effects were generally robust. Funnel plots of publication bias test and forest plot of sensitivity analysis were showed in Figure S2.

#### Hospital stay

**Fig. 4:**
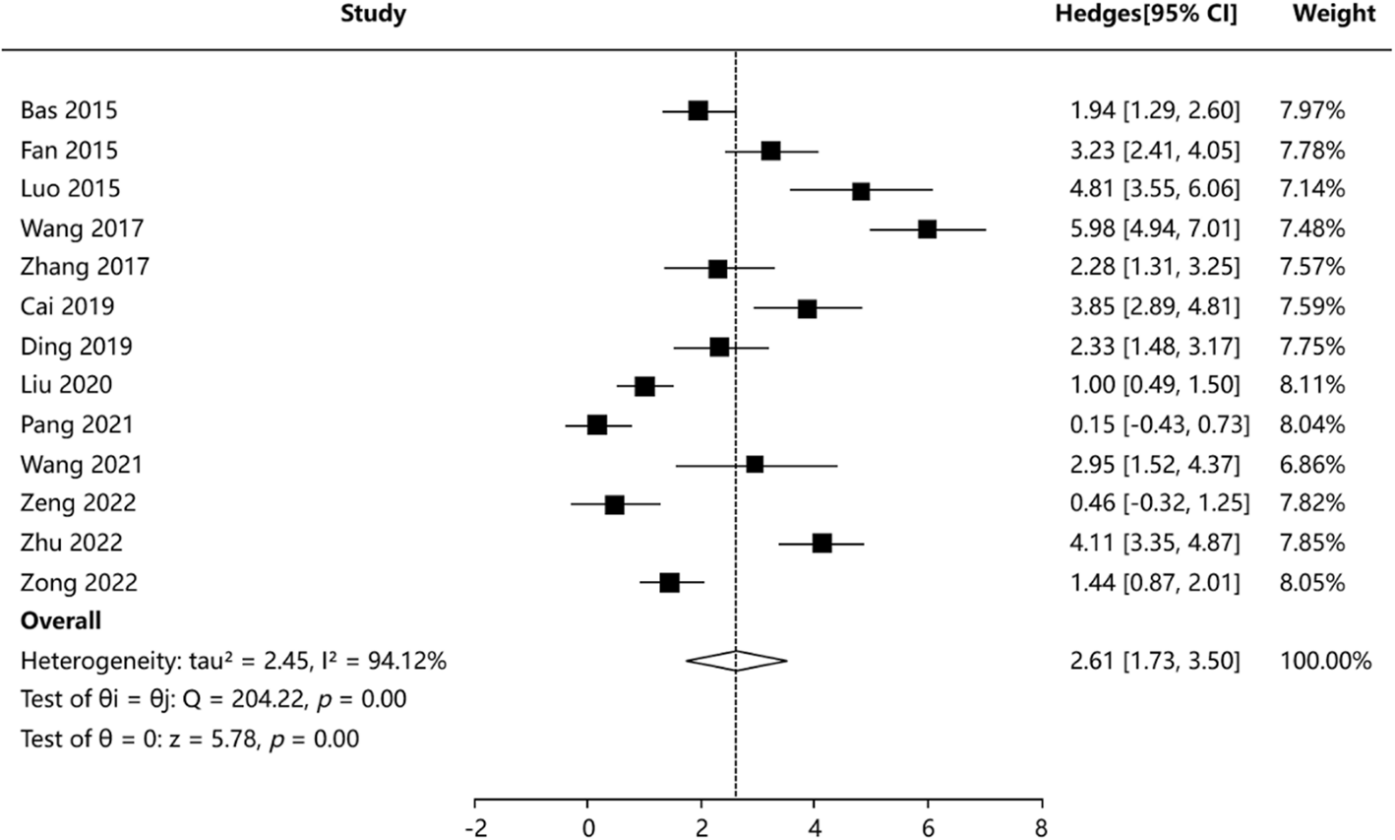
Forest plot showing pooled SMD with 95% CI of hospital stay

Thirteen articles [3, 4, 16–19, 21–27] reported hospital stay, and the results of heterogeneity test were (*P* = 0.000, I^2^ = 94.12%), indicating that the heterogeneity of the included articles was significant. Random-effects model was applied for meta-analysis. The results showed that there was a statistically significant difference in postoperative hospital stay between PCNL group and FURL group: SMD = 2.611, 95%CI: (1.726, 3.496), *Z* = 5.784, *P* = 0.000 (Fig. 4). A statistically significant difference may indicate that postoperative hospital stay was shorter in the FURL group than in the PCNL group. Publication bias test including Egger’s test *P* = 0.008, Begg’s test *P* = 0.020, indicating potential publication bias. We utilized Trim’s method to adjust combined effect value. The combined effect value adjusted by Trim’s method is 95%CI:(0.389,2.387). The above conclusion still holds true. Sensitivity analysis results indicated that the results for pooled effects were generally robust. Funnel plots of publication bias test and forest plot of sensitivity analysis were showed in Figure S3.

#### Complication rate

**Fig. 5:**
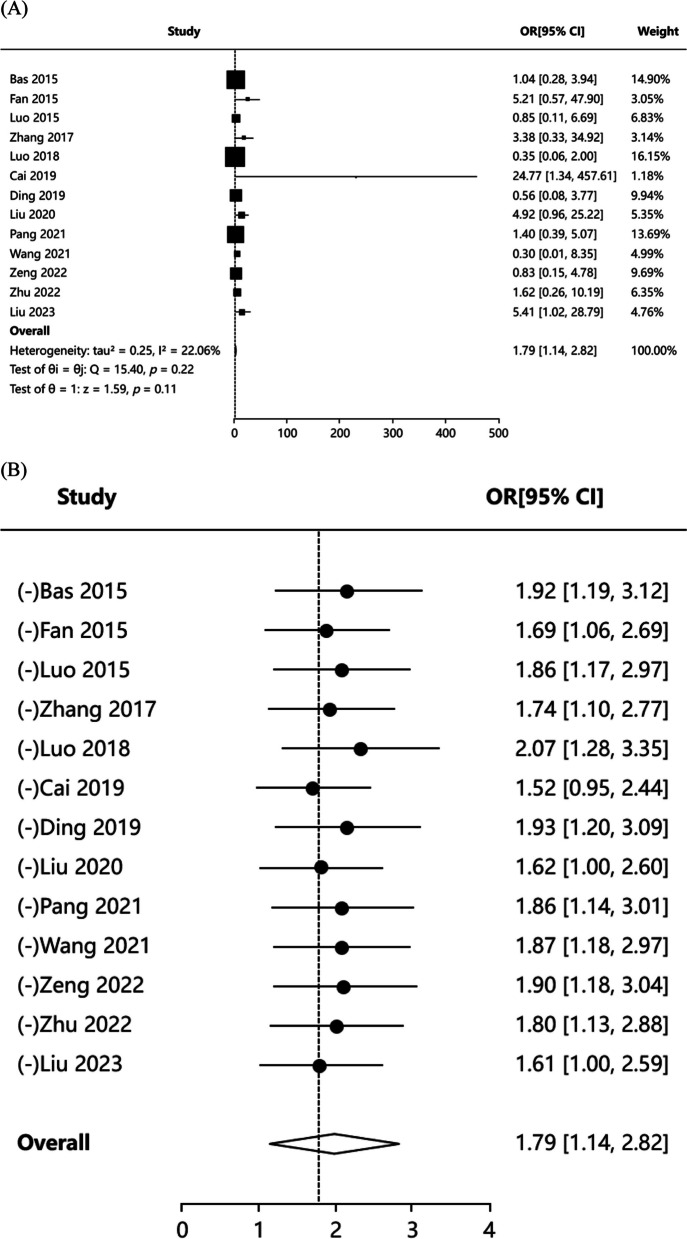
Forest plot showing pooled OR with 95% CI of complication rate. **A** Forest plot of complication rate; **B** Forest plot of sensitive analysis

Thirteen articles [3, 4, 16, 17, 19–26, 28] reported the incidence of postoperative complications in patients, and the results of literature heterogeneity test were (*P* = 0.220, I^2^ = 22.06%), indicating that the heterogeneity of included articles was low, and fixed-effects model was applied for meta-analysis. The results showed that [OR = 1.793,95%CI: (1.139, 2.822), *Z* = 1.586, *P* = 0.113] (Fig. 5A). We found that the significance of OR was inconsistent with the significance of *P* values, which may be due to the relatively large heterogeneity of a study. Publication bias test including Egger’s test *P* = 0.577, Begg’s test *P* = 0.714, indicating no publication bias. The combined effect value adjusted by Trim’s method is 95%CI:(0.952,2.602). So we ended up using Trim's method corrected results. Therefore, the results showed that there was no statistically significant difference in complication rate between PCNL group and FURL group. Sensitivity analysis results indicated that the results for pooled effects were not robust. The sensitivity analysis results also confirm the pooled effect we have previously obtained (Fig. 5B). Funnel plots of publication bias test were showed in Figure S4.

#### Stone-free rate

**Fig. 6:**
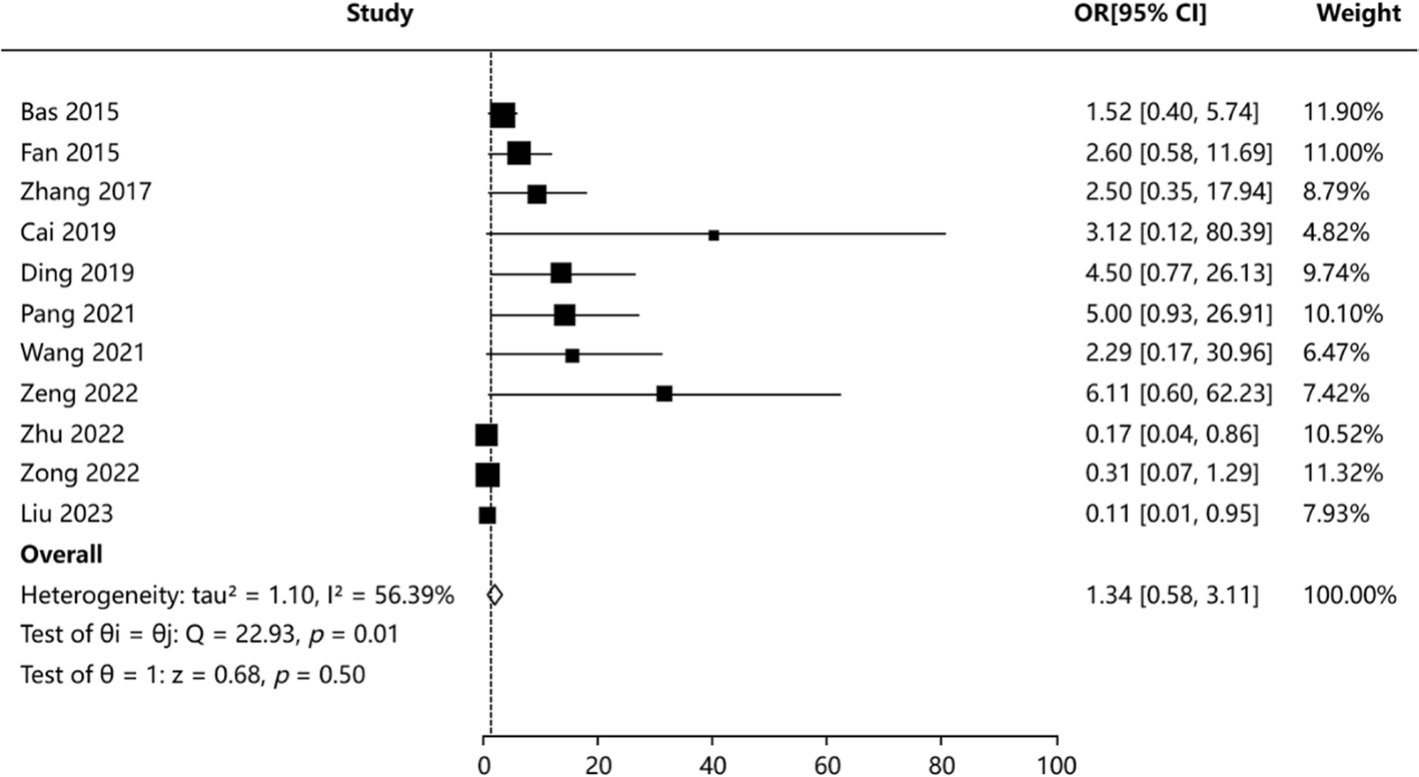
Forest plot showing pooled OR with 95% CI of stone-free rate

Eleven articles [3, 4, 16, 19, 21, 22, 24–28] reported the stone-free rate in patients, and the results of literature heterogeneity test were (*P* = 0.011, I^2^ = 56.39%), indicating that the heterogeneity of the included articles was significant. Random-effects model was applied for meta-analysis. The results showed that there was no statistically significant difference in stone-free rate between PCNL and FURL groups: OR = 1.339, 95%CI: (0.576, 3.112), *Z* = 0.678, *P* = 0.497 (Fig. 6). Publication bias test including Egger’s test *P* = 0.576, Begg’s test *P* = 0.815, indicating no publication bias. The combined effect value adjusted by Trim’s method is 95%CI:(0.522,2.670). The above conclusion still holds true. Sensitivity analysis results indicated that the results for pooled effects were generally robust. Funnel plots of publication bias test and forest plot of sensitivity analysis were showed in Figure S5.

#### Symptom-free rate

**Fig. 7:**
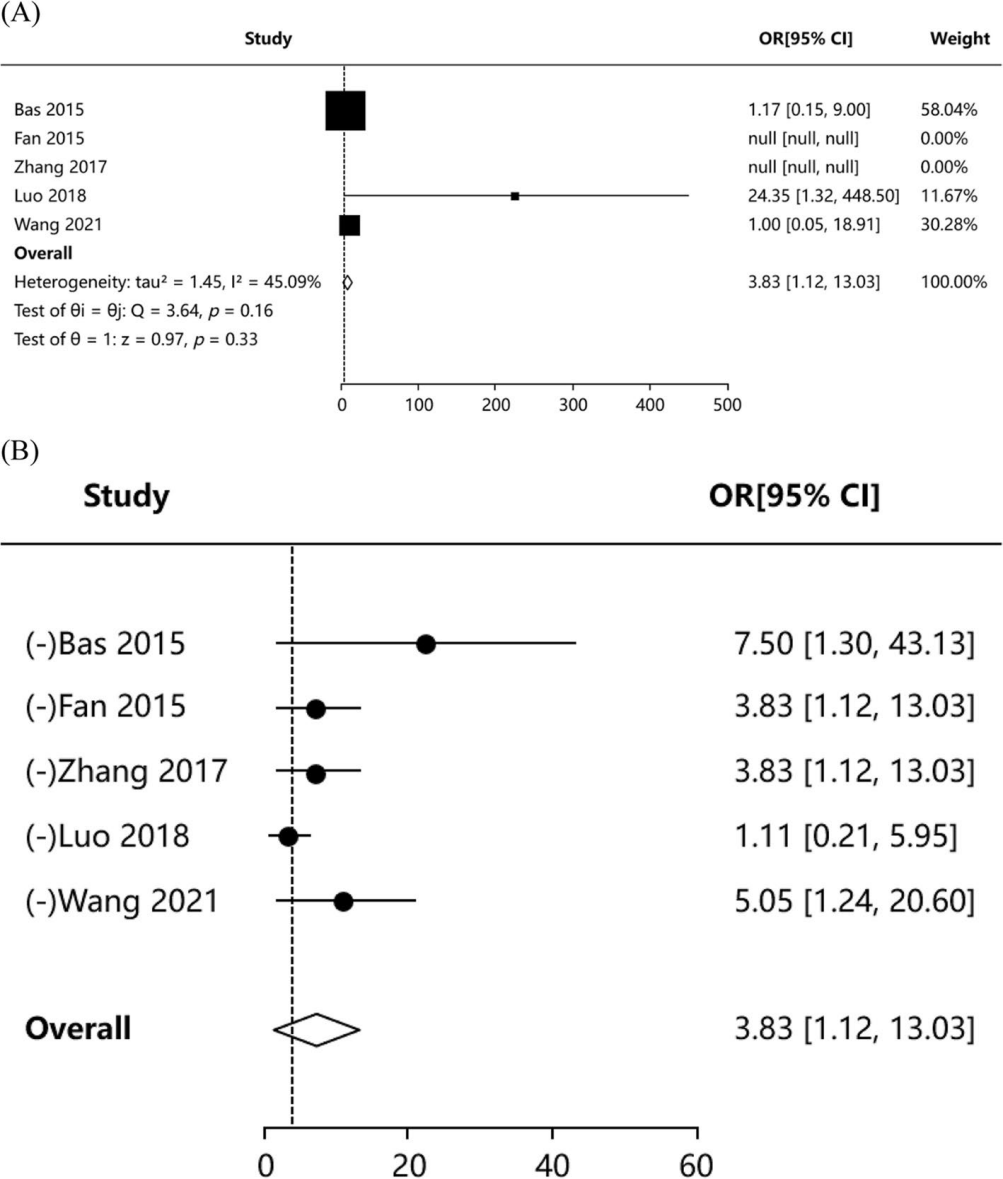
Forest plot showing pooled OR with 95% CI of symptom-free rate. **A** Forest plot of symptom-free rate; **B** Forest plot of sensitive analysis

Five articles [3, 16, 19, 20, 25] reported the symptom-free rate in patients, and the results of literature heterogeneity test were (*P* = 0.162, I^2^ = 45.09%), indicating that the heterogeneity of included articles was low, and fixed-effects model was applied for meta-analysis. The results showed that [OR = 3.826,95%CI: (1.124, 13.026), *Z* = 0.966, *P* = 0.334] (Fig. 7A). We found that the significance of OR was inconsistent with the significance of *P* values, which may be due to the relatively large heterogeneity of a study. Publication bias test including Egger’s test *P* = 0.648, Begg’s test *P* = 0.602, indicating no publication bias. The combined effect value adjusted by Trim’s method is 95%CI:(0.561,10.238). So we ended up using Trim's method corrected results. Therefore, the results showed that there was no statistically significant difference in symptom-free rate between PCNL group and FURL group. Sensitivity analysis results indicated that the results for pooled effects were not robust. The sensitivity analysis results also confirm the pooled effect we have previously obtained (Fig. 7B). Funnel plots of publication bias test were showed in Figure S6.

## Discussion

Our meta-analysis found no significant differences between PCNL and FURL in operating time, complication rate, stone-free rate and symptom-free rate, FURL was superior to PCNL in intraoperative blood loss and postoperative hospital stay. Therefore, FURL may be superior to PCNL in terms of surgical safety and injury to patients. Although PCNL is traditionally considered to be superior to FURL in the treatment of calyceal diverticulum calculi, our study did not find statistical differences in stone-free rate and symptom-free rate between the two groups. In our study, we note that most of the post-2022 articles tend to assume that the efficacy measures of FURL have gradually approached those of traditional PCNL, and the trend in stone-free rate in these studies cannot be ignored. Stone-free rate is the most important indicator of the effectiveness of both procedures in patients with calyceal diverticulum calculi, and with the inclusion of some newer article, our conclusions differ significantly from those of previous meta-analyses [29, 30].

Since Eshghi first reported the use of PCNL for the treatment of calyceal diverticulum calculi in 1987, the success rate, lithotripsy effect and complication rate have improved and developed over time. With the wide application of PCNL, it has gradually become the main minimally invasive surgical method for the treatment of calyceal diverticulum calculi. It has been reported that PCNL treatment of calyceal diverticulum calculi has a stone-free rate of 87.5%−100%, diverticulum closure rate of 76%−100%, and more than 90% of patients can relieve symptoms after follow-up [31, 32]. PCNL can also treat calyceal diverticulum simultaneously with removing diverticulum calculi. Common methods include mechanical dilation of the diverticulum neck and cauterization of the diverticulum mucosa, which help to facilitate closure of the diverticulum [33, 34]. However, PCNL requires puncture to access the surgical site, which inevitably increases injury to the patient, and if the stone is located in the upper pole or ventral side of the kidney, the puncture position and angle also have the risk of causing serious complications [22]. The management of calyceal diverticulum calculi, particularly in the upper pole, presents unique challenges. While lower pole calyceal diverticulum calculi are often associated with a low spontaneous passage rate post-RIRS, upper pole stones introduce additional considerations. The anatomical position of the upper pole can complicate PCNL procedures, imposing limitations on the surgical approach. The access to the upper pole through PCNL may require more complex positioning and puncture routes, increasing the technical difficulty of the procedure. This, in turn, can contribute to a higher risk of complications such as bleeding, adjacent organ injury, and incomplete stone clearance. Furthermore, the angle and approach required for accessing the upper pole can be constrained by patient anatomy, potentially necessitating additional instruments or advanced techniques to achieve successful outcomes. Moreover, the proximity of the upper pole to critical structures such as the ribs and diaphragm can elevate the likelihood of pleural or pulmonary complications, particularly if the puncture trajectory is not carefully planned and executed. Therefore, the risk of these complications is an important factor when considering PCNL for upper pole diverticular stones. Some studies have also shown that the development of visual puncture assisted PCNL technology has improved the safety of puncture process in recent years [35]. Perhaps with the progress and development of traditional PCNL technology, PCNL can not only ensure better curative effect, but also improve the safety of surgery. There is little research on PCNL technology development, and researchers should pay attention to this issue.

FURL has the advantages of less trauma, faster recovery [36]. In theory, FURL can treat diverticulum calculi and diverticulum necks at various locations [37]. Sejiny et al. [38] used FURL to treat 38 cases of calyceal diverticulum calculi. The stone clearance rate was 81.6%, and the asymptomatic rate was 90%. Auge BK et al. [12] also showed that FURL can avoid larger surgical scars for special types of patients, such as pilots, young women and pregnant women. Zhang et al. [39] reported that the success rate of surgery can reach 81.3% or more. FURL directly enters renal pelvis and renal calyces through natural channels of human body, without renal puncture operation, which reduces stress reaction caused by surgical trauma, thus reducing postoperative inflammatory reaction to a certain extent. Zong et al. [27] showed that serum IL-10, CRP and IL-6 levels were increased in patients with both types of surgery on the first postoperative day, but the indexes of FURL patients were significantly lower than those of PCNL patients. The serum BUN, SCr and Cys-C levels of FURL patients were significantly lower than those of PCNL patients on the 7th postoperative day. The holmium laser fiber used in FURL is relatively slim. This would increase the space available between the ureteroscope and the ureter or access sheath, thus increasing irrigation outflow, obtaining good vision and reducing intrarenal pressure [27]. FURL also has certain limitations, and FURL treatment of calyceal stones is limited by the size of the stone and the position of the neck of the calyceal [40]. The original concept was that flexible ureteroscopy was not suitable for long or thin diverticulum neck, and flexible ureteroscopy was longer for stones larger than 2 cm in diameter, and the one-time stone clearance rate was low, usually requiring two operations. However, there is a case of FURL surgery that have not been published on scientific research platform in 2023, which successfully treat type IV calyceal diverticulum (complete occlusion of diverticulum orifice) stones, providing a new idea for FURL to overcome some limited types and treat special types of calyceal diverticulum calculi. The difficulty of FURL lies in locating diverticulum and managing diverticulum stones in the lower calyx [41], which can be solved by intraoperative localization with color Doppler ultrasound or by injecting methylene blue into the collecting system through a ureteral catheter or flexible ureteroscope [42]. With the gradual development of FURL technology and the improvement of doctors’ experience in FURL, FURL may break through some bottlenecks in the near future and realize qualitative leap at the technical level.

In terms of comparison between PCNL and FURL, the indications for the two procedures are different. PCNL is best used for calyceal diverticulum stones located in the posterior middle pole and provides the opportunity for direct ablation of diverticulum, identification of diverticulum openings, and further management of diverticulum are relatively simple. FURL as a minimally invasive surgical method may be more suitable for the treatment of calyceal diverticulum calculi located in the anterior middle pole, but the difficulty in identifying the opening and the low ablation rate should be considered. At present, the best surgical method for the treatment of calyceal diverticulum calculi is still controversial. Both operations have their own unique indications, advantages and disadvantages. With the progress and development of technology and more and more studies on the efficacy and safety of the two new surgical techniques, the best surgical method for the treatment of calyceal diverticulum calculi may be determined in the future. Urologists who treat calyceal diverticulum calculi should continue to pay attention to it.

From a scientific and rigorous point of view, our meta-analysis study also has certain limitations. First, although multiple databases were searched for this study, the number of articles retrieved was limited. Second, the stone location, stone burden, diverticulum neck anatomy and other conditions of patients are different, the level and proficiency of operators are not consistent, the surgical equipment models of different hospitals and different postoperative treatment programs bring inevitable bias to this study. Third, since it is now clearly believed that passive dilation may significantly reduce operative time for FURL, ultrasound assistance and appropriate operative positioning may significantly reduce operative time for PCNL. Therefore, standardization of surgical equipment, surgical procedures, and patient conditions may make the results more convincing for comparisons of variable parameters such as operating time and postoperative hospital stay. Due to the limitations of some original studies, we could not include only articles using the same surgical equipment, the same surgical procedure, and patient-specific stone locations in the study design when conducting the secondary study. Therefore, the comparison of such variable parameters in our study can only be used as a reference, and the impact of some indicators that cannot be standardized on the robustness of the results cannot be ignored. Since not all RIRS procedures are performed with the same device, and not all PCNL procedures have the same criteria, coupled with the limitations of the original study, it is likely that the bias in our study caused by the inability to fully standardize the two procedures will not be completely resolved. This bias masks individual differences between different surgical procedures and changes in specific situations, so the results and conclusions drawn from our study may not be universally applicable. The diversity of different devices and surgical standards may make the advantages or risks in some specific situations invisible, which affects the reliability and generalizability of our study results to some extent. Fourth, as FURL technology advances, there is a tendency for surgical selection to be biased towards FURL, and the selection bias that may have existed in this original study cannot be resolved by our secondary study. Fifth, only English and Chinese literatures were retrieved from the database, and some non-English literatures were not included in the study, resulting in selection bias.

## Conclusion

In conclusion, our meta-analysis found that FURL showed a trend toward potentially superior clinical safety to PCNL, and that FURL tended to approach PCNL in terms of surgical efficacy as the technology evolved. However, due to the inevitable bias caused by the incomplete standardization of surgical procedures in our study, whether FURL is indeed superior to PCNL in terms of safety, whether the efficacy of FURL is really close to PCNL, and whether FURL can surpass PCNL as the first choice for the treatment of calyceal diverticular calculi in the future need to be further verified by multi-center, large-sample and high-quality studies.

## Supplementary Information

Supplementary Material 1.

Supplementary Material 2.

Supplementary Material 3.

Supplementary Material 4.

Supplementary Material 5.

Supplementary Material 6.

## Data Availability

All the data generated or analyzed during this study are included in the published article.

## Abbreviations

PCNL: Percutaneous nephrolithotomy
FURL: Flexible ureteroscopy
CD: Calyceal diverticulum
ESWL: Extracorporeal shock wave lithotripsy
SCR: Stone clearance rate
NOS: Newcastle-Ottawa Scale
SMD: Standard mean difference
OR: Odds ratio
CI: Confidence interval
PRISMA: Preferred Reporting Item of the Guidelines for Systematic Review and Meta-Analysis
