# Supplementary material for: Percutaneous nephroscopy versus flexible ureteroscopy in the treatment of calyceal diverticulum calculi: a meta-analysis
Source: BMC Urol. 2025 Jan 2;25:1. doi: 10.1186/s12894-024-01655-w (PMC11694468; doi:10.1186/s12894-024-01655-w)
Supplement: Supplementary file 2 — Supplementary Material 2. [file 12894_2024_1655_MOESM2_ESM.pdf]

(A)

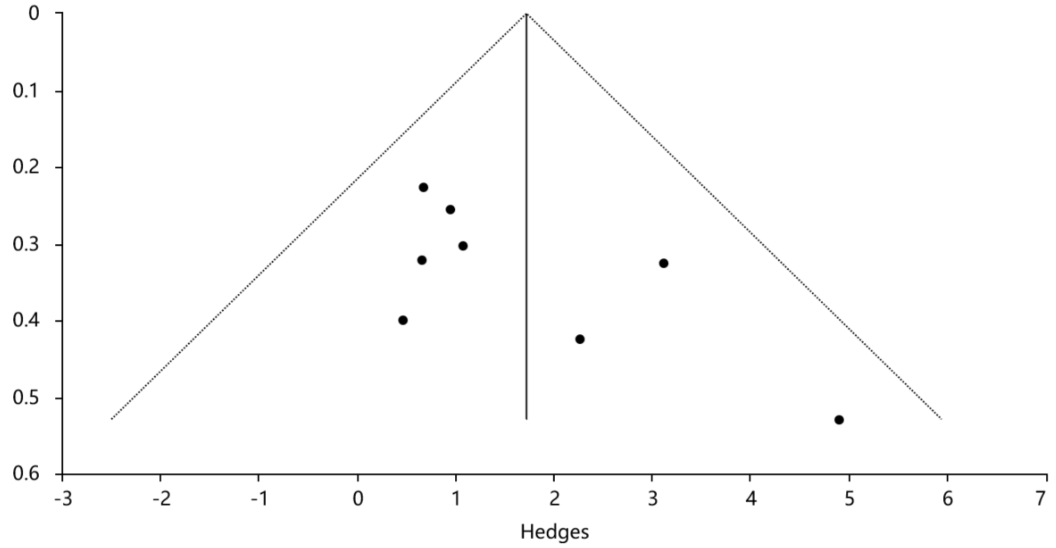
0
0.1
0.2
0.3
0.4
0.5
0.6
-3
-2
-1
0
1
2
3
4
5
6
7
Hedges

(B)

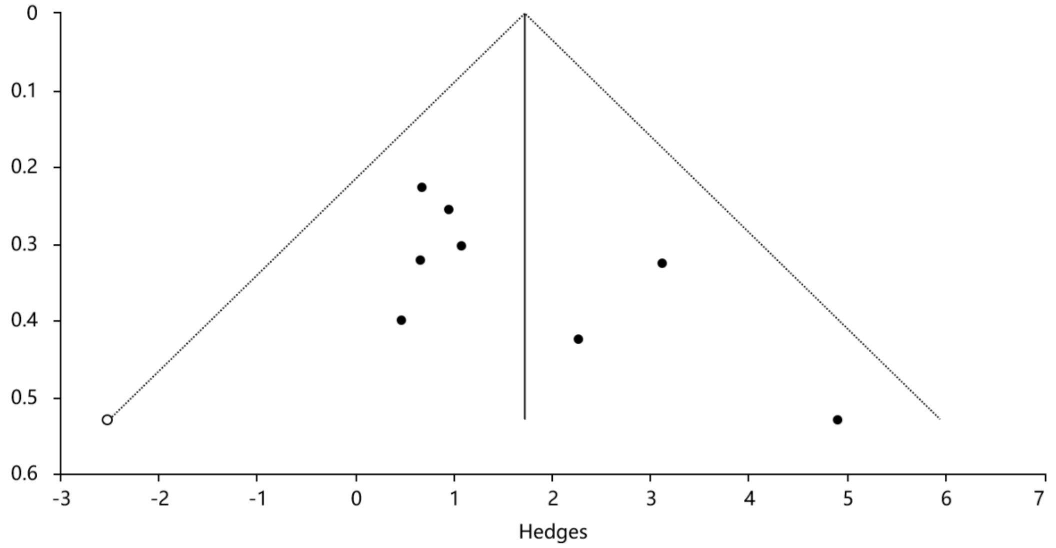
0
0.1
0.2
0.3
0.4
0.5
0.6
-3
-2
-1
0
1
2
3
4
5
6
7
Hedges

(C)

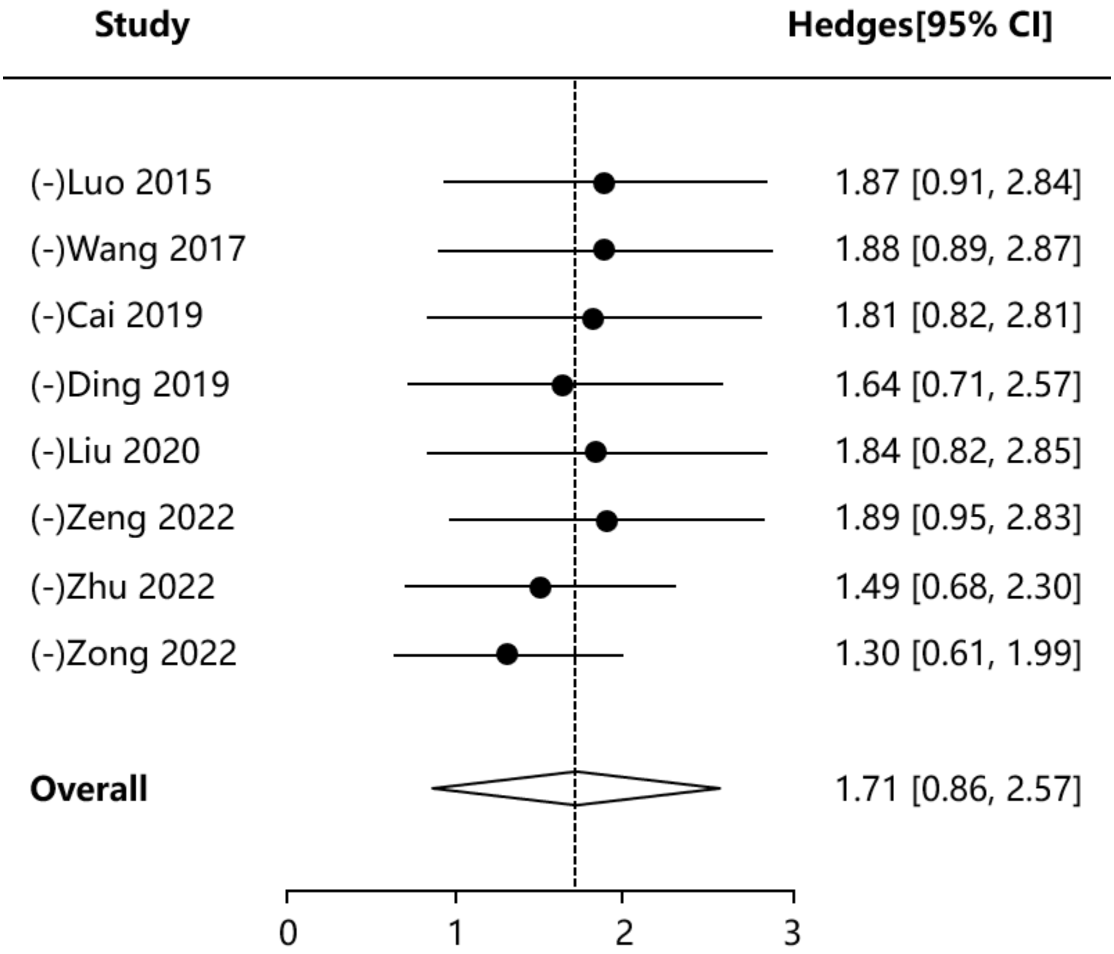

Figure S2 Funnel plots of publication bias test and forest plot of sensitivity analysis (blood loss)

(A) Funnel plot of publication bias test. (B) Funnel plot of Trim's method. (C) Forest plot of sensitivity analysis.
