# Supplementary material for: Percutaneous nephroscopy versus flexible ureteroscopy in the treatment of calyceal diverticulum calculi: a meta-analysis
Source: BMC Urol. 2025 Jan 2;25:1. doi: 10.1186/s12894-024-01655-w (PMC11694468; doi:10.1186/s12894-024-01655-w)
Supplement: Supplementary file 5 — Supplementary Material 5. [file 12894_2024_1655_MOESM5_ESM.pdf]

(A)

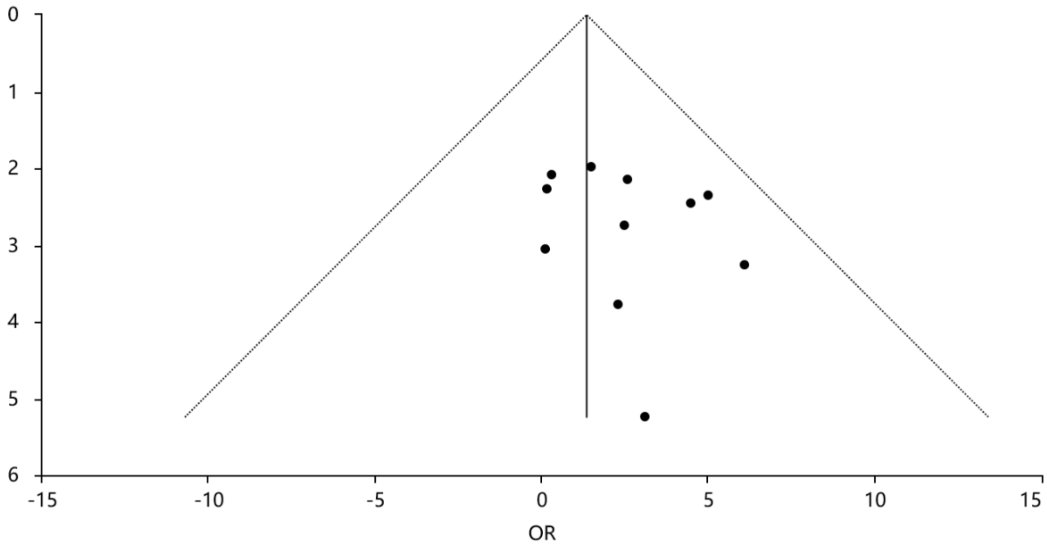

(B)

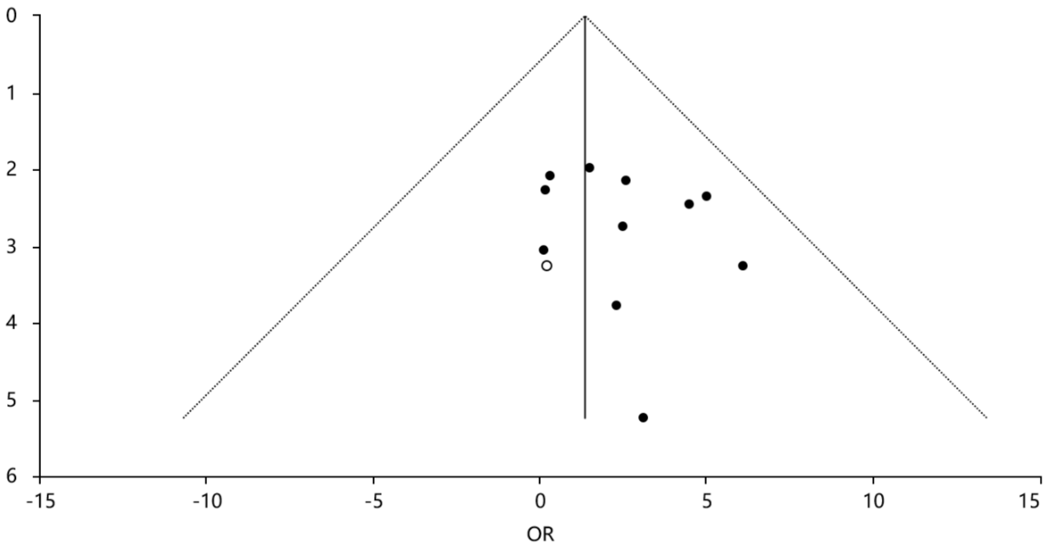

(C)

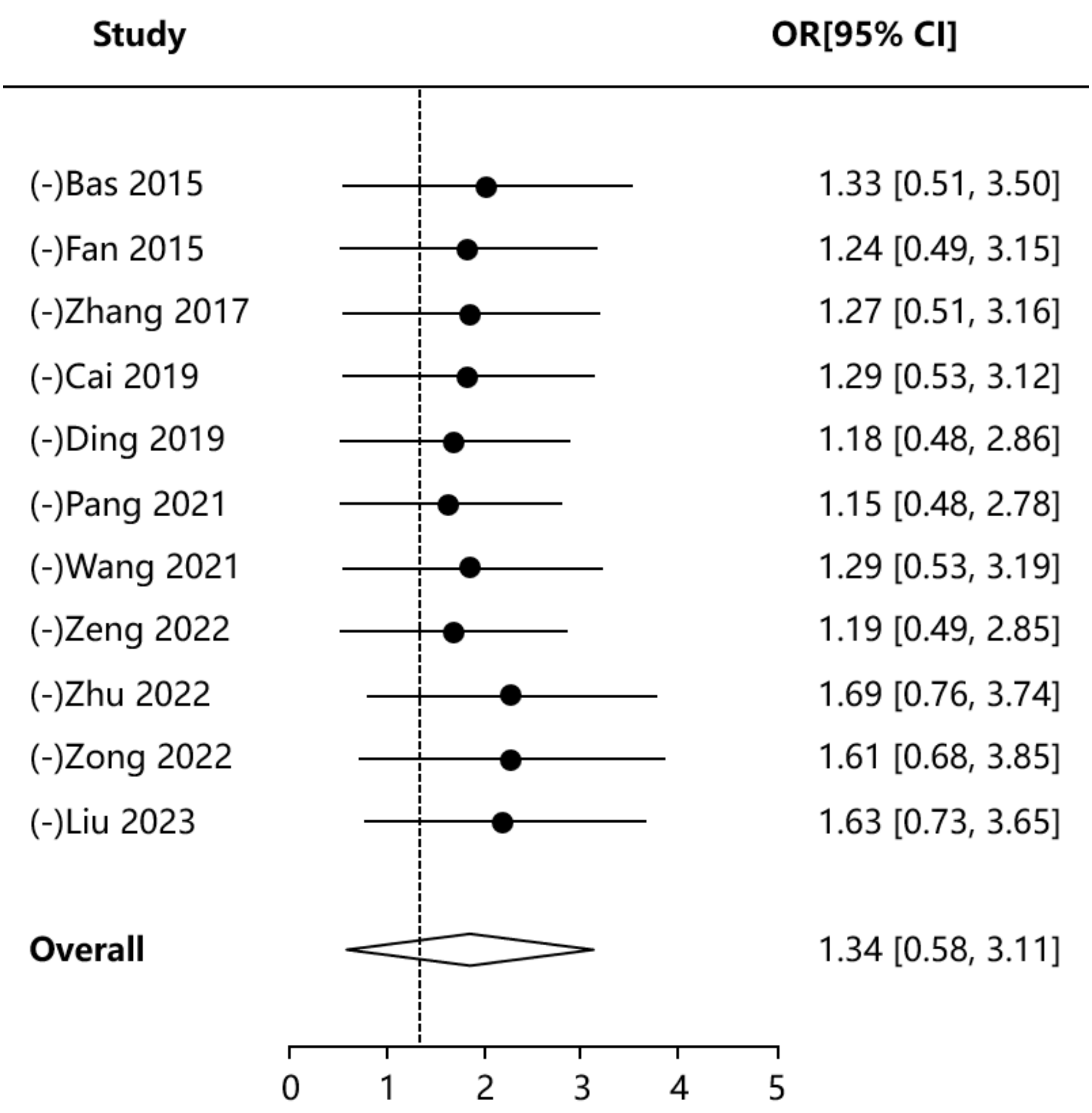

Figure S5 Funnel plots of publication bias test and forest plot of sensitivity analysis (stone-free rate)

(A) Funnel plot of publication bias test. (B) Funnel plot of Trim's method. (C) Forest plot of sensitivity analysis.
